# Age-related decline of online visuomotor adaptation: a combined effect of deteriorations of motor anticipation and execution

**DOI:** 10.3389/fnagi.2023.1147079

**Published:** 2023-06-20

**Authors:** Na Li, Junsheng Liu, Yong Xie, Weidong Ji, Zhongting Chen

**Affiliations:** ^1^Shanghai Changning Mental Health Center, Shanghai, China; ^2^Shanghai Key Laboratory of Brain Functional Genomics, Affiliated Mental Health Center, School of Psychology and Cognitive Science, East China Normal University, Shanghai, China; ^3^Key Laboratory of Space Active Opto-Electronics Technology, Shanghai Institute of Technical Physics, Chinese Academy of Sciences, Shanghai, China

**Keywords:** aging, visuomotor adaptation, eye movement, manual tracking, online control

## Abstract

The literature has established that the capability of visuomotor adaptation decreases with aging. However, the underlying mechanisms of this decline are yet to be fully understood. The current study addressed this issue by examining how aging affected visuomotor adaptation in a continuous manual tracking task with delayed visual feedback. To distinguish separate contributions of the declined capability of motor anticipation and deterioration of motor execution to this age-related decline, we recorded and analyzed participants' manual tracking performances and their eye movements during tracking. Twenty-nine older people and twenty-three young adults (control group) participated in this experiment. The results showed that the age-related decline of visuomotor adaptation was strongly linked to degraded performance in predictive pursuit eye movement, indicating that declined capability motor anticipation with aging had critical influences on the age-related decline of visuomotor adaptation. Additionally, deterioration of motor execution, measured by random error after controlling for the lag between target and cursor, was found to have an independent contribution to the decline of visuomotor adaptation. Taking these findings together, we see a picture that the age-related decline of visuomotor adaptation is a joint effect of the declined capability of motor anticipation and the deterioration of motor execution with aging.

## Introduction

The capacity of the sensorimotor system for adapting to environmental changes is essential for the interaction between individuals and the world. But this fundamental function appears to decline with aging. Several empirical studies have shown that older people, compared to younger ones, have poorer performances of adaptation to perturbed visual input during visuomotor control in both ballistic reaching tasks (Buch et al., 2003; Bock, 2005; Bock and Girgenrath, 2006; Seidler, 2006; Heuer and Hegele, 2008; Vandevoorde and de Xivry, 2019; Wolpe et al., 2020; Li et al., 2021) and online tracking tasks (Bock and Schneider, 2002; Teulings et al., 2002). However, it is still unclear what mechanisms in the visuomotor loop underlie the aging effect on visuomotor adaptation. The present study aims to address this question by comparing older and young people's eye–hand coordination in a manual tracking task with delayed visual feedback.

Declines in older people's motor control can be broadly classified into two types based on their behavioral and neural features and neural basis: declines in the capability of motor anticipation and deterioration of motor execution. Motor anticipation refers to the ability to predict the course/trajectory of dynamic visual events and corresponding behavioral responses (Kandel et al., 2000). This process involves adapting the mapping between the stimulus and one's internal dynamic representation of ongoing events and is related to a broad neural network including the premotor cortex, basal ganglia, anterior cingulate, posterior medial parietal area, superior parietal-occipital cortex, and middle intraparietal sulcus (Adam et al., 2003; Glover, 2004 for review; Glover et al., 2012). This process becomes less precise as people age, leading to difficulties in anticipating coming action accurately (Diersch et al., 2016). Moreover, the capability of motor anticipation is tightly related to general cognitive functions (Varghese et al., 2016; Svoboda and Li, 2018; Chen et al., 2022), which also decline with the increase in age (Salthouse, 1996; Raz, 2000; Park et al., 2003). Our previous study (Li et al., 2021) found that the decline in age-related visuomotor adaptation was mediated by cognitive decline with aging, suggesting a potential relationship between aging effects on the decline of visuomotor adaptation and impairment of motor anticipation. This possibility was further examined in the present study.

Another possible cause for the aging effect on motor adaptation is the deterioration of motor execution. Motor execution is usually defined as the online control processes from the initiation of the response to the completion of the movement. This course needs humans to integrate outside and internal information to monitor and calibrate the movements online (Woodworth, 1899; Elliott et al., 2001; Glover, 2004; Liu et al., 2008). This process is mainly related to the primary motor cortex, cerebellum, supramarginal gyrus, and superior parietal lobule (Glover et al., 2012). In most cases, motor movements become slower and less accurate with aging. Movement times of older adults were longer than young adults by 26–69%, even for simple reaching movements (Stelmach et al., 1988; Amrhein et al., 1991; Pohl et al., 1996; Walker et al., 1997). Exceptionally, older performers sometimes attempt to do something as quickly as young people, but with lower accuracy. For example, multiple studies have shown that times of ballistic shots during visuomotor adaptation show no difference between older and young adults, but the accuracy of rapid hitting is lower for older adults (Huang et al., 2018; Li et al., 2021). In these cases, the slowness with aging was possibly a learned adaptive strategy to cope with declined motor execution function (Lamb et al., 2016). Since visuomotor adaptation was generally measured by movement accuracy and/or movement delay in most studies (e.g., Bock and Schneider, 2002; Teulings et al., 2002; Buch et al., 2003; Bock, 2005; Bock and Girgenrath, 2006; Seidler, 2006; Heuer and Hegele, 2008; Vandevoorde and de Xivry, 2019; Wolpe et al., 2020; Li et al., 2021), it is also possible that the observed aging effect of visuomotor adaptation is a consequence of deteriorated motor execution of older people.

However, it is difficult to determine from the existing literature which mechanism, declined capability of motor anticipation or deterioration of motor execution, is responsible for the decline of visuomotor adaptation with aging. A major line of evidence for declined visuomotor adaptation with aging is from ballistic reaching tasks with visual perturbation. This type of motor task requires individuals to move a cursor in an out-and-back trajectory, hitting the target and then returning to the center. Motor errors caused by perturbation are incrementally reduced across trials, indicating performers' adaptation to visual perturbations. Multiple studies (Buch et al., 2003; Bock, 2005; Bock and Girgenrath, 2006; Seidler, 2006; Heuer and Hegele, 2008; Vandevoorde and de Xivry, 2019; Wolpe et al., 2020; Li et al., 2021) have shown that older people have poorer adaptation to visual perturbation than young people. But it is difficult to use results from these tasks to distinguish the mechanisms of the decline of adaptation because of the ballistic movements in these tasks. On each trial, people only perform one shot which is the result of both motor anticipation and motor execution at a single moment, making it difficult to distinguish their separate contributions to visuomotor adaptation. Regarding such difficulty, the present study turned from ballistic reaching to manual tracking as an alternative approach to investigating the mechanisms of the aging effect on visuomotor adaptation.

Manual tracking, another type of motor task, requires people to control a moving object (e.g., cursor) in the center of a dynamic target and calibrate errors between the target and cursor (Jagacinski, 1977; Foulkes and Miall, 2000; Miall and Jackson, 2006). In these tasks, visuomotor adaptation was examined by measuring how performers coped with perturbation of delaying the cursor feedback, which often occurs in teleoperations in practice (Gerisch et al., 2013; Alvarez-Aguirre et al., 2014; Khasawneh et al., 2019). To deal with such perturbation, young people tend to advance the position of the cursor to reduce the viewed target–cursor displacement. Older people also use similar strategies of advancing the target position, but to a substantially reduced extent, indicating declined visuomotor adaptation of older people (Jagacinski et al., 1993; Bock and Schneider, 2001; Teulings et al., 2002; see also in Bock and Schneider, 2002). In contrast to ballistic movements where motor anticipation and execution are hardly distinguished through one-shot movement, control of manual tracking is a real-time task, in which performers need to continuously make online adjustments in response to the target motion and this real-time nature allows for distinguishing those mechanisms by observing the continuous changes of different behavioral indicators. Regarding the current study, we attempted to analyze the eye movements and lag-corrected random error of visuomotor control, respectively, of the individual performer during manual tracking. Different from previous studies using ballistic reaching tasks, the introduction of manual tracking with delayed feedback in the current study would help to separately measure the contributions of declined motor anticipation and deterioration of motor execution to the age-related decline of visuomotor adaptation, thus further investigating and distinguishing potential underlying mechanisms of the aging effect on visuomotor adaptation.

In addition, we introduced the method of eye movement tracking in the current study to evaluate individuals' motor anticipation, as mentioned above. Previous literature has demonstrated that eye movements are tightly related to anticipated motion trajectory. For example, gaze movements usually precede movements of the cursor or arm during visuomotor control (Campbell and Wurtz, 1978; Koken and Erkelens, 1992; Kandel et al., 2000; Elliott et al., 2001; Liu et al., 2008; Huang and Hwang, 2012; Niehorster et al., 2015; Danion and Flanagan, 2018). Studies on smooth pursuit eye movements have indicated that such preceding eye movements reflect people's prediction of the future trajectory of the moving object (Kettner et al., 1997; Barnes, 2008; Kowler et al., 2019), and help to enhance motion prediction in manual interception (Bennett et al., 2010; Spering et al., 2011; see review in Fooken et al., 2021). Even monkeys' eye movements indicate a short-term prediction of future motions of the target in a tracking task (Kettner et al., 1997). Studies using reaching tasks (Ariff et al., 2002; Rand and Rentsch, 2016; Brouwer et al., 2018) have also found similar preceding eye movements before hand movement, which reflects individual participants' cognitive strategies and motor planning adjustment during visuomotor adaptation. All these findings consistently indicate that eye movements leak information about motor anticipation. On the other hand, multiple studies have shown that the execution of eye movements per se is not substantially affected by aging (Bock et al., 2014; Huang et al., 2017). Based on both lines of evidence, we chose to use anticipatory preceding eye movements to index the individual differences, especially the age-related differences, of motor anticipation during manual tracking in the current study.

In addition to analyzing anticipatory preceding eye movements, we also compared the random errors of the tracking performances between older people and young adults, after controlling for the lag between the target and the cursor. We hypothesized that this difference represented the potential aging effect on motor execution, for most of the aging effect on motor anticipation was expected to be excluded when the lag between the target and the cursor was corrected. However, please note that the increase in this random error still might be due to multiple factors, including a worse correction to visual feedback, worse movement selection, and worse simple movement execution. We further discussed these possibilities in the Discussion section.

In short, we conducted a manual tracking task with a 200-ms visual feedback delay for both older and young adults in the current study. During the task, we recorded both the manual trajectory and the eye movements, using the latter as an index of motor anticipation (measured by gaze-target lag). We also calculated the remaining motor error after accounting for the target-cursor lag effect, as a measure of motor execution. As a brief preview of the results, we found that older people had significantly worse adaptation to perturbations (i.e., visual feedback delay), and, interestingly, old people's performances in visuomotor adaptation had a considerable correlation (*r* = −0.744) with their gaze-target lags, indicating the importance of motor anticipation in visuomotor adaptation and the impact of aging on it. However, even after controlling for the differences in the baseline performances and motor anticipation between the two groups, older participants still showed worse performances in the adaptation phase than young people, indicating that the age-related decline in visuomotor adaptation was also partially caused by declined motor execution of older people.

## Methods

### Participants

Fifty-six participants in total volunteered for the current study. Data from one young participant and three older participants were excluded before analysis because the recording of their eye movement was too noisy and missing in some trials. Twenty-nine healthy older participants (range: 60–73 years, mean: 65.97 years, SD: 3.8 years, 13 women) with twenty-three healthy young participants (range: 19–27 years, mean: 22.93 years, SD: 1.97 years, 11 women), as a control group, were finally included for data analysis. A χ^2^ test showed that the sex ratio of neither group was significantly different from 1:1 (older people: *p* = 0.576; younger adults: *p* = 0.835). The handedness of the participants was checked with Edinburgh Handedness Inventory (Oldfield, 1971) to ensure that all the participants were right-handed. Visual acuity was measured for all the participants to confirm normal or corrected-to-normal vision. No participant had a history of neurological diseases, psychiatric disorders, or musculoskeletal dysfunctions. Each participant was paid 80 RMB for their participation. The study was approved by and conformed to the standards of the Human Research Ethics Committee for Non-Clinical Faculties at East China Normal University.

### Apparatus

**Figure 1:**
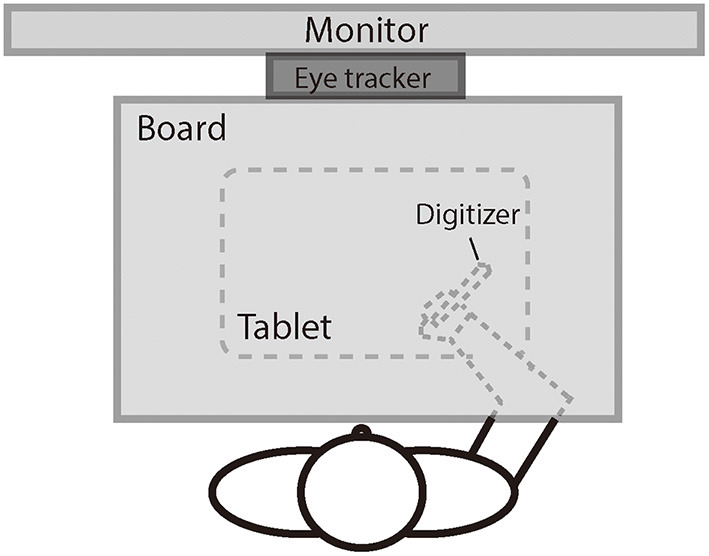
The illustration of the experiment setting.

Figure 1 illustrates the experimental setup. The participant was seated comfortably in a dim room facing an LED monitor (ASUS VG278, 1920 × 1080 pixels, 27 inches, 60 Hz) positioned in the frontal plane 50 cm from the participant's eyes. Head movements were restrained by a chin rest, ensuring that the eyes were directed toward the center of the screen. A board was positioned under the participant's chin to keep the hand out of sight. The participant controlled the cursor (a 1° × 1° red dot) on the screen using a digitizer with the right hand. The scales of the hand movement and the cursor movement were physically matched. The digitizer was restrained on a slider so that the participant could only move the digitizer leftward or rightward and not lift it. The movements of the left eye were recorded at a sampling rate of 1,000 Hz using the remote mode of Eyelink Portable Duo eye movement tracker (SR Research, Mississauga, ON, Canada). We chose to track only one eye to keep a higher sampling rate (i.e., 1,000 Hz) for better analysis of pursuit. The left eye was chosen because our laboratory setting has the infrared camera of the tracker better aligned to the left eye than to the right eye.

Hand movement trajectories were sampled at a rate of 40 Hz using a digitizer and a tablet (152 × 95 mm, Wacom, Intuos). The experiment was programmed in MATLAB with the Psychtoolbox package (Brainard, 1997; Pelli, 1997; Kleiner et al., 2007).

### Task and procedure

**Figure 2:**
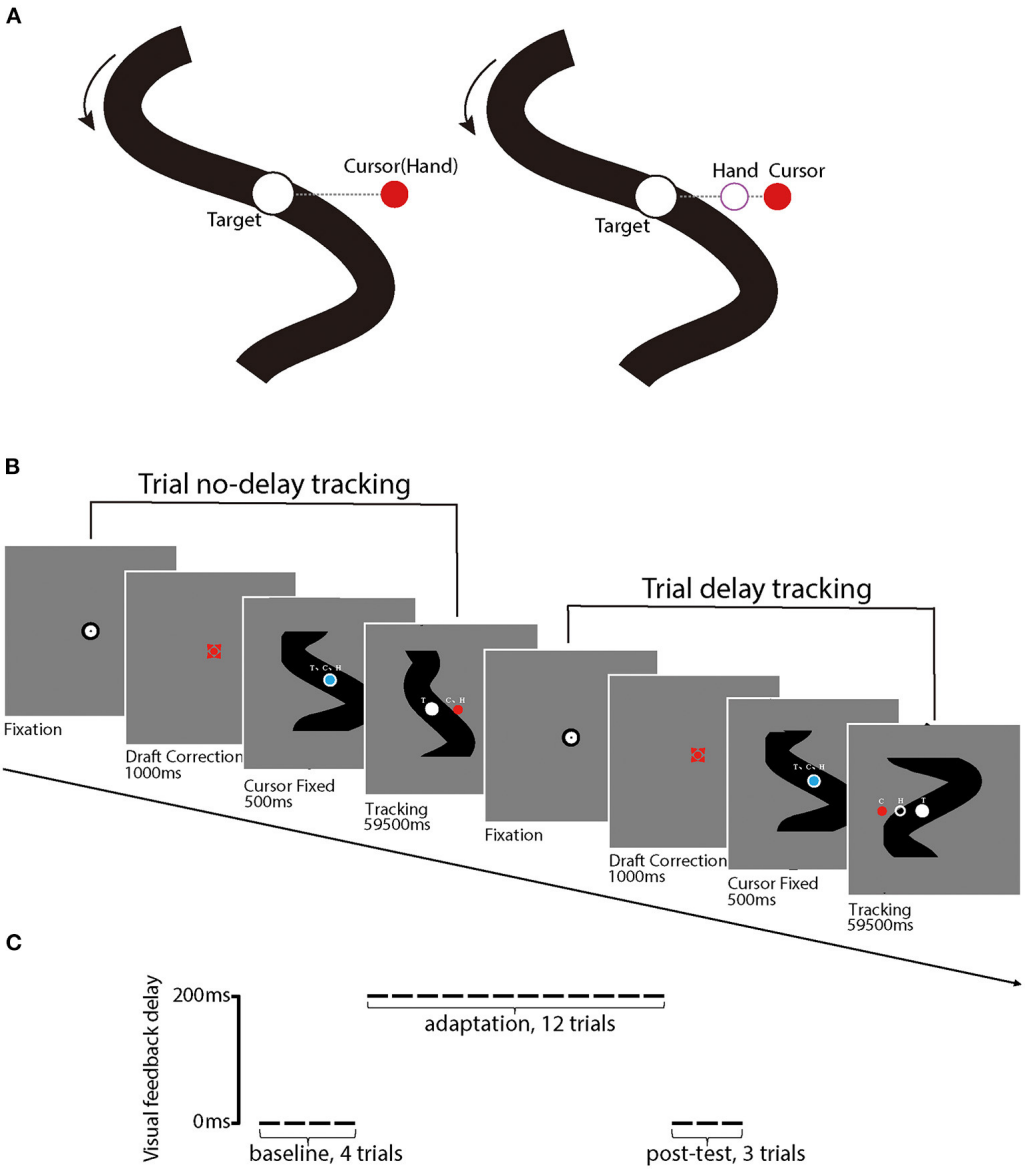
**(A)** illustrates stimuli used in the experiment, including the red point that indexes the position of the cursor (C) and the white circular target (T). Black solid curves are target movement trajectories that are visible to participants; the dashed line (invisible for participants) represents the distance between the cursor and the target. The left panel illustrates a trial without delay perturbation, in which the hand position (H) and C position are matched. In contrast, the right panel illustrates a trial with delay perturbation, in which the *C* position was behind the *H* position. **(B)** illustrates the procedures of a sample trial without delay perturbation (first) and a sample trial with (second), respectively. **(C)** illustrates the experiment design for each participant, including the first 4 baseline trials, 12 delay adaptation trials, and 3 post-test trials. Delay perturbation was an additional 200 ms artificial feedback delay in reference to the actual hand movement direction in the delay adaptation phase.

Each participant performed a manual-tracking task adapted from Rohde et al. (2014). On each trial, the participant controlled a red cursor point (diameter: 1° of visual angle) using the digitizer to track a white target circle (diameter: 1.4° of visual angle) on a gray background. The participant was required to keep the cursor stay inside of the target circle during the whole trial. The target moved horizontally across the screen along an unpredictable trajectory which was constructed by the addition of five nonharmonic sine waves with frequencies of 0.09, 0.165, 0.195, 0.375, and 0.495 Hz and amplitudes of 20, 50, 20, 100, and 20 pixels, with phases randomly determined for each trial. The maximum shift of trajectory was limited to 12.9° of visual angle (i.e., 387 pixels) along the x-axis; the trajectory would be re-generated if the maximum shift surpassed the limit. The target trajectory was shown in the background at the beginning of each trial and moved from top to bottom, while it was kept visible for motor planning and visual feedback during the manual tracking (see Figure 2). We also uploaded a video clip that records the procedure of a sample trial (available at https://osf.io/tq8ye/q8ye/).

For the first 500 ms of each trial, both the cursor (blue) and the target (white) were presented and kept stationary in the center of the screen, with the cursor upon the target. Then, the target started to move and the cursor turned from blue to red, as a signal for the start of the tracking procedure. After the tracking procedure, which lasted 59,500 ms, the participant moved the digitizer back to the start position (i.e., the center of the screen). The next trial would not start until the participant placed the cursor into the start region. The hand and eye movement trajectories were tracked and recorded online by the digitizer and Eyelink Portable Duo, respectively, throughout the whole tracking procedure of each trial.

Each participant first completed four practice trials without perturbation, each of which lasted 42 s, and then started the formal experiment. In the formal experiment, the participant sequentially received 4 baseline trials, 12 perturbation adaptation trials, and 3 post-test trials, each of which lasted 60 s. In the baseline and the post-test phases, the red cursor veridically reflected the trajectory of the digitizer. In the perturbation phase, the movement trajectory of the cursor was delayed by 200 ms. All the participants reported that the delay was noticeable after the experiment. This delay was sufficiently small to trigger a delay adaptation (Cunningham et al., 2001; Rohde et al., 2014).

### Data analysis

The hand movement trajectory and the eye movement were recorded online at a sampling rate of 40 Hz and 1,000 Hz, respectively. We first excluded the beginning 2 s of each formal trial from analysis to avoid the effects of the initial transient response (Bock, 2005; Li et al., 2005, 2006, 2011; Niehorster et al., 2013). We then normalized the cursor position data from 40 Hz to 1000 Hz by interpolation to match the hand movement and gaze data on the same scale for analysis. For each trial, the hand movement data were low-pass filtered at 10 Hz using a Butterworth filter implemented in MATLAB. We then calculated the root mean square error (RMSE) between the target and the cursor and computed the lags among the target, the cursor, and the gaze using cross-correlation techniques (Gerisch et al., 2013). A positive lag indicates that the cursor moves behind the target and vice versa.

The gaze data were first low-pass filtered using a second-order recursive Butterworth filter with a cutoff frequency of 50 Hz. We adopted an algorithm originally developed by Nyström and Holmqvist (2010) that uses gaze velocity to identify saccade. First, we used the Savitzky–Golay smoothing filter (Savitzky and Golay, 1964) to get the velocity and acceleration measurements from the gaze coordinates. We then removed the events in which eyes were closed or the records were physiologically impossible (velocity over 1,000°/s or acceleration over 100,000°/s^2^). Then, we estimated the velocity peak for each trial. We calculated the average and standard deviation of all samples with velocities lower than a given initial peak velocity detection threshold of 200°/s, then updated the threshold as a new threshold which was equivalent to the last average plus six times the standard deviation for each iteration. The final velocity peak threshold was confirmed until the absolute value between two adjacent iterations was smaller than 1°/s. Finally, we detected two types of eye movements, saccades and glissade movements, which were wobbling movements at the end of many saccades (Weber and Daroff, 1972; Flierman et al., 2019). Saccade onset and offset were identified by searching backward and forward for the stop criterion from each detected saccade peak. Specifically, saccade onset was defined as the first sample that goes below all samples' average plus three times standard deviation, and where it was monotonically decreasing. Saccade offset was defined as a weighted combination of the velocity at saccade onset and a locally adaptive noise factor (the current saccade samples' average plus three times standard deviation), and where it was monotonically increasing. Glissade movements started from the offset of the preceding saccade and continued until the data monotonically increased after the last velocity peak sample. We counted the times of saccades and glissades together and then removed them from the gaze data to prevent their intervention in the analysis of pursuit eye movement trajectories. To compensate for the removal of these data, we filled the gaze data using a method based on linear interpolation.

## Results

**Figure 3:**
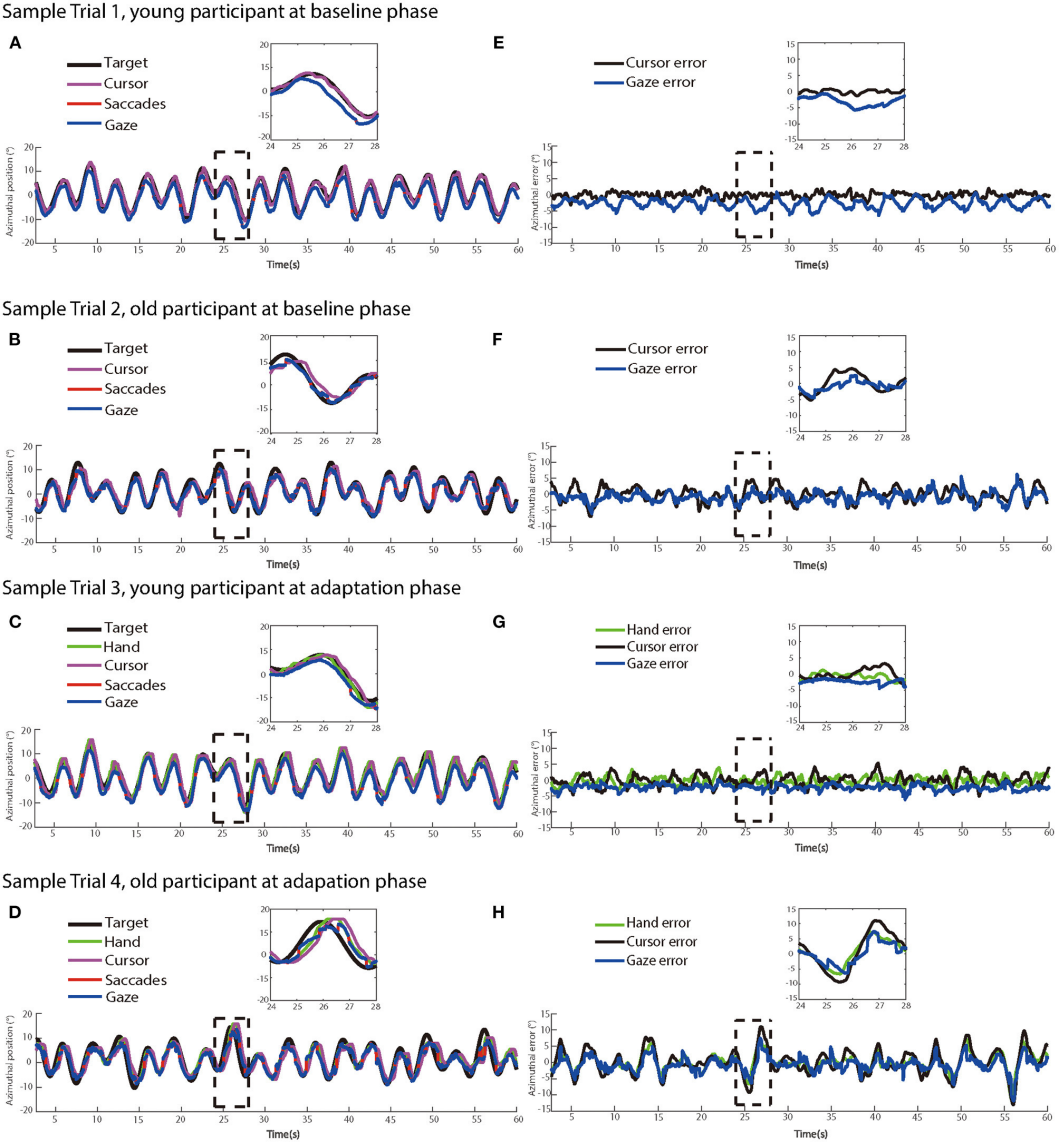
The left panels show dynamics of target, hand, cursor, saccade, and eye movement positions in two sample trials at **(A)** the baseline phase and **(C)** the adaptation phase from a young participant, and two sample trials at **(B)** at the baseline phase and **(D)** the adaptation phase from an older participant, respectively. **(E–H)** The right panels show the time series of target-hand errors, target-cursor errors, and target-eye errors of the same trials at the baseline phase and the adaptation phase from the same participants, respectively.

Figure 3 illustrates recorded manual trajectories and gazes and corresponding time series of errors performed by two sample participants from different age groups in four sample trials of no-delay and delay experimental phases, respectively. In the no-delay trials, manual and eye-tracking paths were close to the target trajectory. In contrast, a feedback delay of 200 ms was introduced in the adaptation phase and this manipulation increased the offset between manual tracking paths and the target trajectory, indicating an increased delay from target movement to manual tracking. In addition, this offset appeared to be larger in the sample trial by the older participant (Figures 3D, H) than one by the young participant (Figures 3C, G).

### Manual tracking performance

**Figure 4:**
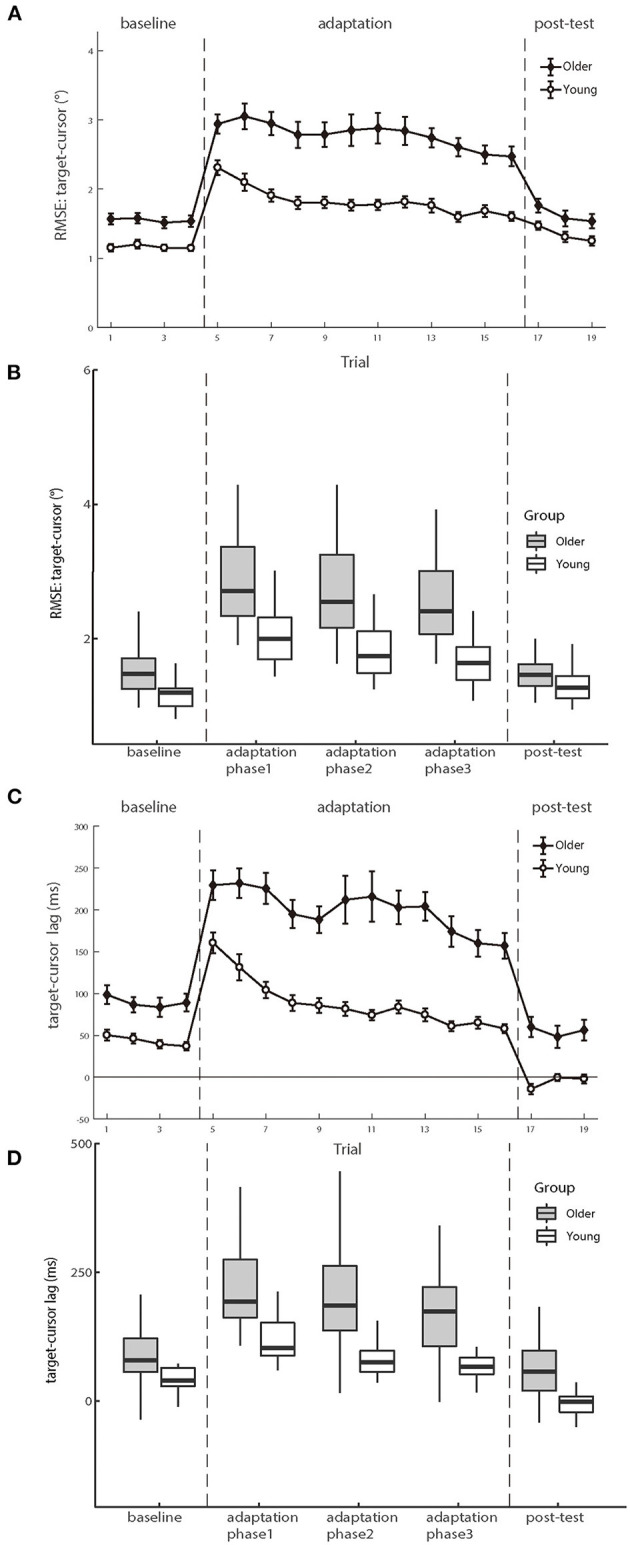
**(A, B)** illustrate the means of RMSE and **(C, D)** illustrate the mean lag between target and cursor as a function of trial number for the groups of young and older participants, respectively. Positive lag represents that the cursor lags behind the target. Error bars depict ±1 standard errors of means.

Figure 4 shows the mean RMSEs and lags between the target and cursor as a function of trial number for two age groups, respectively. We first analyzed the mean RMSE between the target and cursor to investigate whether the older and young participants had differences in the manual tracking task. The test phase condition was divided into baseline, adaptation phase1, adaptation phase2, adaptation phase3, and post-test in the calculation. There were significant main effects of age group [*F* (1.50) = 22.747, *p* < 0.001, *η_p_^2^* = 0.313] and test phase [*F* (4.200) = 103.604, *p* < 0.001, *η_p_^2^* = 0.674], as well as a significant interaction between age group and test phase [*F* (4.200) = 12.338, *p* < 0.001, *η_p_^2^* = 0.198]. Please see the full results from Supplementary Table A1.

For the baseline phase, a mixed-design ANOVA showed that there was a significant group effect [*F* (1.50) = 16.089, *p* < 0.001, *η_p_^2^* = 0.243], indicating that cursor tracking position error for older adults (1.55 ± 0.08°) was larger than young adults (1.16 ± 0.05°) even without delay. Over the adaptation period, the cursor's movement always showed 200 ms slower than the hand's position, consequentially introducing more tracking errors. After controlling for the baseline difference, a significant difference was still found on the RMSE between the two groups in the adaptation trials [young adults 0.66 ± 0.06° vs. older adults 1.23 ± 0.12°, *F* (1.50) = 15.692, *p* < 0.001, *η_p_^2^* = 0.239], indicating that the feedback perturbation made a larger decrease in movement control accuracy for older adults than young adults.

Existing literature has reported that older adults have worse adaptation to feedback perturbation than young adults during manual tracking, reflected by more increase in *target-cursor lag* by perturbation (Welford, 1958; Braune and Wickens, 1985; Jagacinski et al., 1993). Our results replicated such findings. In the baseline phase, a repeated measure ANOVA revealed that the lag between older and young groups had a significant difference [*F* (1.50) = 13.954, *p* < 0.001, *η_p_^2^* = 0.218)], and the cursor lagged behind the target by 89.466 ms (±10.397 ms *s.e*.) in the older group, whereas by 43.261 ms (±4.522 ms *s.e*.) in the young group. After controlling the baseline performance, a significant difference was still found between the two groups in the adaptation trials [*F* (1.50) = 22.483, *p* < 0.001, *η_p_^2^* = 0.310], with a substantially large difference between the older group (114.014 ± 7.135 ms) and the young group (45.889 ± 7.135 ms). Therefore, both the *RMSE* and the *target-cursor lag* suggested that older adults performed worse and less adaptively than young adults in manual tracking. Please see the full results in Supplementary Table A2.

### Eye movement performance

#### Saccadic eye movements between age groups

**Figure 5:**
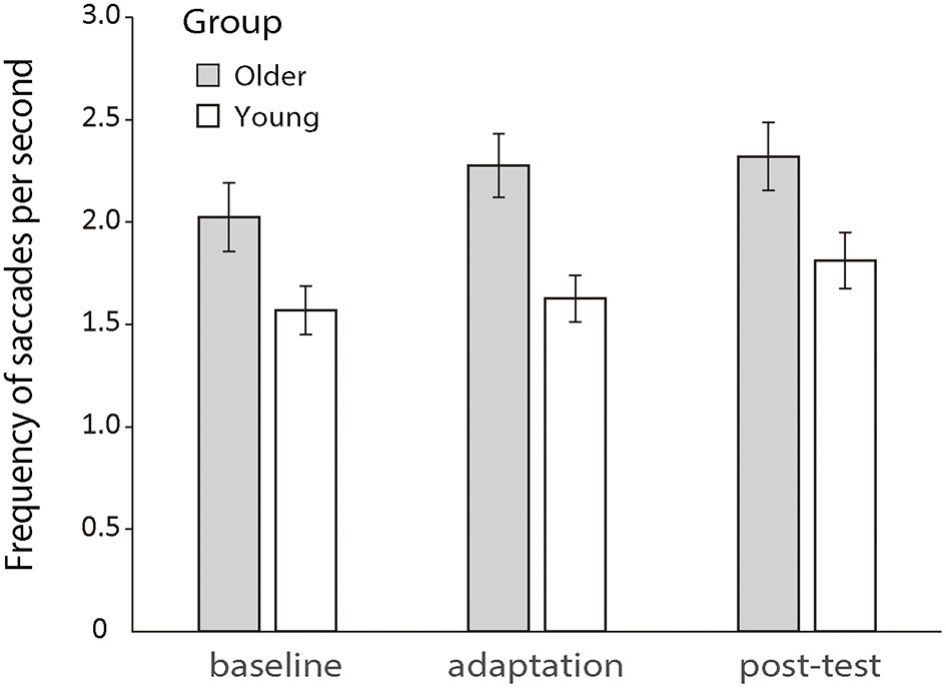
Mean frequencies of saccades per second during the baseline, adaptation, and post-test phases for the two groups, respectively. Error bars depict ±1 standard errors of means.

Previous literature has shown that increased saccadic frequency is usually correlated to poorer cognitive processing and attentional functions (Kimmig et al., 2001; Galna et al., 2012; Stuart et al., 2018). So, here, we first analyzed the saccadic frequency between the two groups during the manual tracking. A 2 (older vs. young group) × 3 (baseline, adaptation, and post-test) repeated-measures ANOVA revealed a significant main effect of groups [*F* (1.50) = 6.968, *p* = 0.011, *η_p_^2^* = 0.122] and phases [*F* (2.100) = 12.686, *p* < 0.001, *η_p_^2^* = 0.202], but no interaction effect was found between groups and phases [*F* (2.100) = 1.751, *p* = 0.179, *η_p_^2^* = 0.034]. Older and young participants both had a rather stable rate of saccades which were about 2.23 and 1.64 per second across all conditions, respectively (see Figure 5).

#### Pupil Size

As documented by multiple studies (Hess and Polt, 1964; Stanners et al., 1979; Murphy et al., 2011; Jerčić et al., 2020), pupil size is an effective measure of the mental load or arousal level of individual participants. In this experiment, we compared the pupil size variations across the trials between groups to investigate whether the procedure of the experiments affected the older and young participants differently in their arousal levels. We first calculated the mean pupil area (unit: pixel) recorded by the eye tracker for each trial and then performed a 2 (older vs. young) × 3 (phases of baseline, adaptation, and post-test) mixed-design ANOVA on the mean pupil areas. The analysis showed that neither the main effect of phase nor the interaction between group and phase was significant [phase: *F* (2.100) =1.501, *p* = 0.228, *η_p_^2^* = 0.029; group × phase: *F* (2.100) = 0.247, *p* = 0.782, *η_p_^2^* = 0.005], indicating that there was no marked change of arousal level for either group during the experiment. The main effect of the group was significant [*F* (1.50) = 6.428, *p* = 0.014, *η_p_^2^* = 0.114]. However, please note that the significant main effect of the group was not necessarily attributed to the actual differences between mean pupil sizes of the two groups considering that we did not record the actual size of pupils but one by pixels (Hayes and Petrov, 2016). The effect could be caused by other alternative factors (e.g., individual differences in the spatial relationship between the tracked eye and the eye tracker camera).

#### Coordination among target, eyes, and hand

**Figure 6:**
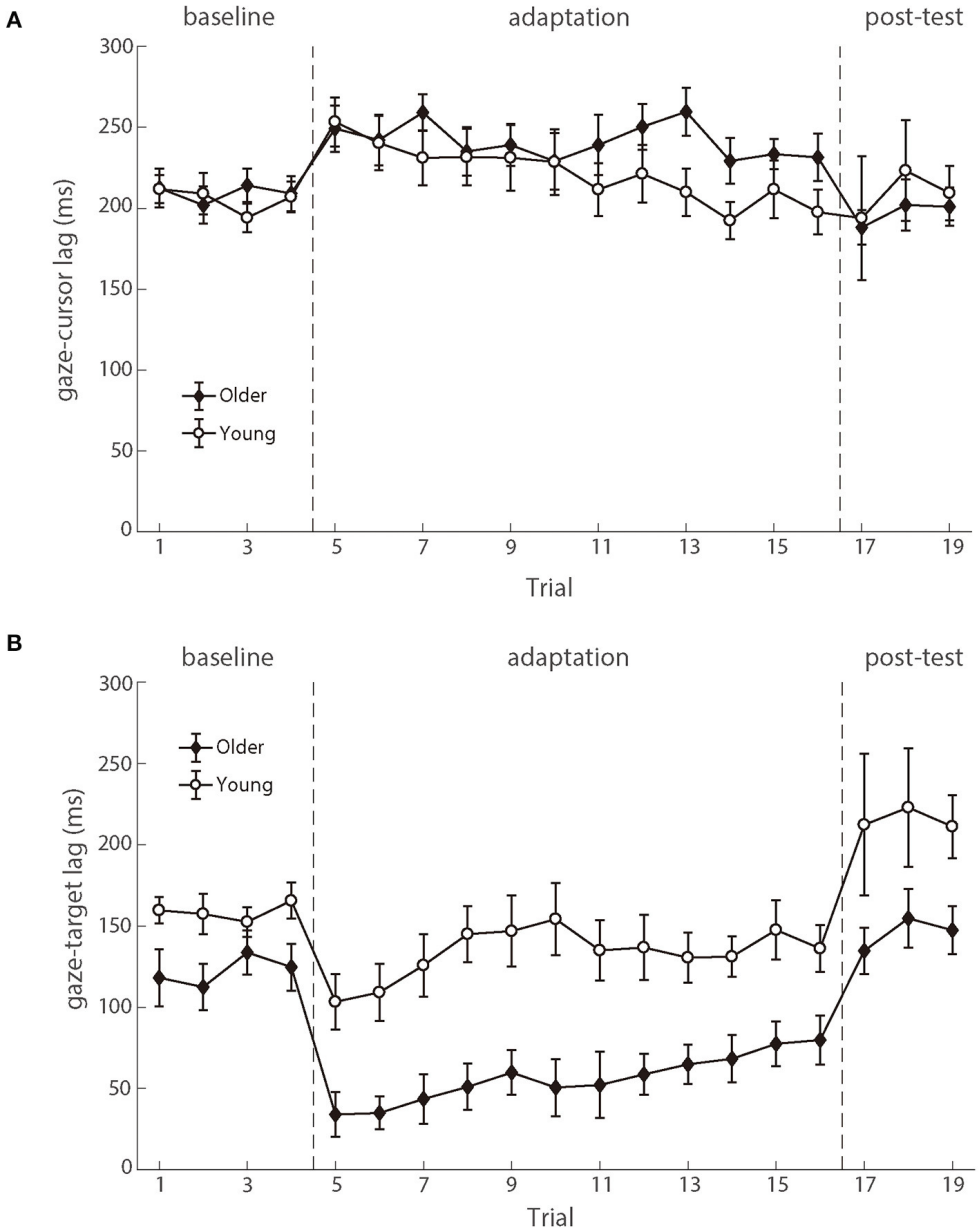
**(A)** illustrates the mean lags between eye and cursor and **(B)** illustrates the mean lags between eye and target as a function of trial number for young participants (white circle) and older participants (black diamond). Error bars depict ±1 standard errors of means.

As shown in Figure 6, the gaze preceded the hand movement for all participants in both the baseline and adaptation phases (Mathew et al., 2019). The analysis on the *gaze-cursor lag* showed a main phase effect, *F* (2.100) = 4.267, *p* = 0.017, *η_p_^2^* = 0.079, and the lag in the baseline phase was shorter than in the adaptation phase [mean difference = −23.852, *p* = 0.006]. Figure 6A illustrated the similar lags between gaze and cursor for both age groups during different phases, and repeated ANOVAs revealed no significant difference between the two groups either during the baseline phase [*F* (1.50) = 0.072, *p* = 0.789, *η_p_^2^* = 0.001] or during the adaptation phase [*F* (1,50) = 0.895, *p* = 0.349, *η_p_^2^* = 0.018]. These results suggested that eye-hand coordination was comparable between the two age groups.

The eye movements also preceded the target, as demonstrated by the positive *gaze-target lag* across the phases (Figure 6). There was a significant main effect of groups [*F* (1.50) = 11.323, *p* = 0.001, *η_p_^2^* = 0.185], as well as the main effect of phases [*F* (2.100) = 27.338, *p* < 0.001, *η_p_^2^* = 0.353]. No significant interaction effect was found. The *gaze-target lag* differed significantly between age groups in the baseline phase, *F* (1.50) = 5.113, *p* = 0.028, *η_p_^2^* = 0.093. After controlling for the baseline difference between the two groups, there was still a significant difference between the two age groups in the adaptation phase, *F* (1.50) = 7.694, *p* = 0.008, *η_p_^2^* = 0.133 (see Figure 6B). In brief, the observed gaze-target coordination showed that the young adults had both better target trajectory prediction and better adaptation to delayed feedback, as compared to the older adults.

**Figure 7:**
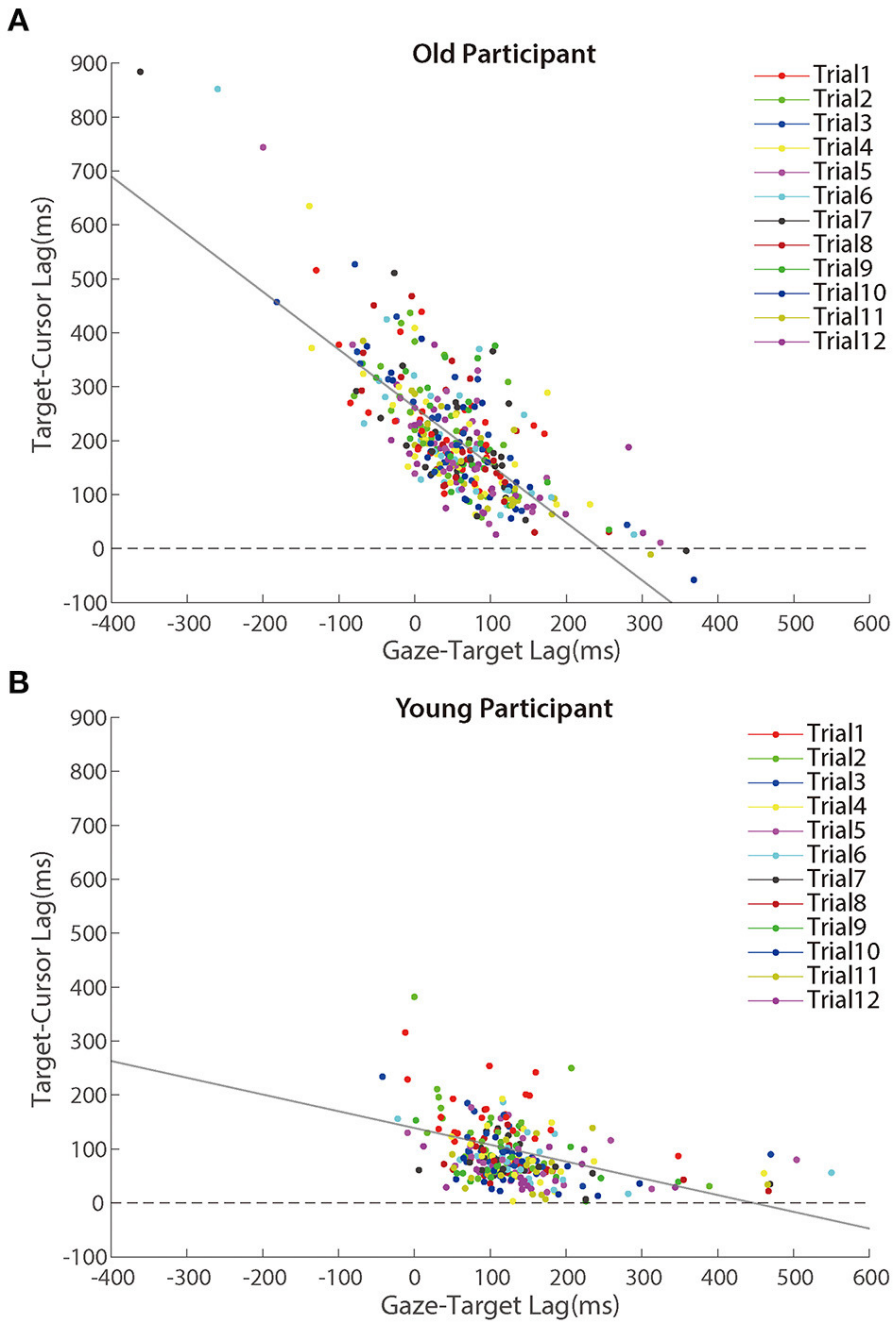
Scatter plots of lags between target and cursor in the adaptation phase for **(A)** older participants and **(B)** young participants. Trials in the same order are marked using a unique color. The solid lines represent simple linear regression model fits.

Further analyses revealed that the correlation between the lag of target-cursor and gaze-target was a significant negative during the adaptation phase (Figure 7). That was to say, eye movement that preceded the target more was accompanied by better cursor tracking to the target. We found significant correlations between age and *target-cursor lag* in older people (*r* (346) = 0.190, *p* < 0.001) and young adults [*r* (274) = 0.110, *p* = 0.067], respectively. Then, we tested the relationship between the *target-cursor lag* and the *gaze-target lag* and found a strong negative correlation in older adults [*r* (346) = −0.744, *p* < 0.001], as well as a mild correlation in young adults [*r* (274) = −0.358, *p* < 0.001]. These correlations indicated similar negative linear relationships between eye movement and hand-tracking performance for young and older adults.

We also performed a linear mixed model to distinguish whether *gaze-target lag* could explain the decreased adaptation with aging in manual tracking. Three alternative models were performed (see Table 1), which all included a control variable, *trial order*, which represented the order of a certain trial and was related to the general learning effect during the adaptation. We then tested two variables, *age* and *gaze-target lag*, which represented the ages of individual participants and measured lags between gaze and target in specific trials, respectively. The model comparison (Table 1) showed that Model 2 which included *gaze-target lag* had significantly better predictions than Model 1, χ^2^ = 321.67, *df* = 1, *p* < 0.001, and the fitting indices also showed a preference for Model 2 over Model 1. These results indicated that *gaze-target lag* was an effective predictor of *target-cursor lag*, even after the factor of individual ages had been controlled. On the other hand, there was no significant prediction difference between Model 2 and Model 3, in which the variable *age* was removed, χ^2^ = 0.29, *df* = 1, *p* = 0.592. Moreover, the fitting indices both showed no preference for Model 2 over Model 3. Combining these findings, we concluded that *gaze-target lag* made a major contribution to the age-related decrease of adaptation in manual tracking.

**Table 1:** Linear mixed model comparison.

**Model**	**df**	**Log Likelihood**	**Deviance**	**AIC**	**BIC**
Model 1: TC_lag ~ age + TO	7	−1982.4	3964.9	3978.9	4005.8
Model 2: TC_lag ~ age + TO + GT_lag	8	−1821.6	3643.2	3659.2	3690.0
Model 3: TC_lag ~ TO + GT_lag	7	−1821.7	3643.5	3657.5	3684.5

AIC, Akaike information criterion; BIC, Bayesian information criterion. TC_lag, *target-cursor* lag; TO, trial order; GT_lag, *gaze-target* lag.

Despite Figure 7 showing a negative correlation between *target-cursor lag* and *gaze-target lag*, this negative correlation could be merely driven by between-participant differences. In other words, within-participant variations of *gaze-target lag* induced by visuomotor adaptation might not contribute to the negative correlation between *target-cursor lag* and *gaze-target lag*. To test this possibility, we removed between-participant variations of *gaze-target lag* and *target-cursor lag* by centralizing them (i.e., subtracting the mean *gaze-target lag* and the mean *target-cursor lag* of each participant) for each participant and re-calculated the correlations. We found that the correlations did not change dramatically for either group [older people: *r* (346) = −0.723, *p* < 0.001; young adults: *r* (274) = −0.458, *p* < 0.001]. We also re-ran Model 3 with centralized *gaze-target lag* and *target-cursor lag*. Note that centralization of *target-cursor lag* (i.e., removal of between-participant variation) would make age as a between-participant variable no more predictive to *target-cursor lag*. So, it was no need to re-run Model 1 and Model 2. The results showed that *gaze-target lag* was still a significant fixed factor after centralization and its coefficient was negative (*b*_GT_lag_ = −0.711, *p* < 0.001). All these findings consistently indicated that within-participant variations of *gaze-target lag* and *target-cursor lag* were negatively correlated with each other.

### Random error of visuomotor control

**Figure 8:**
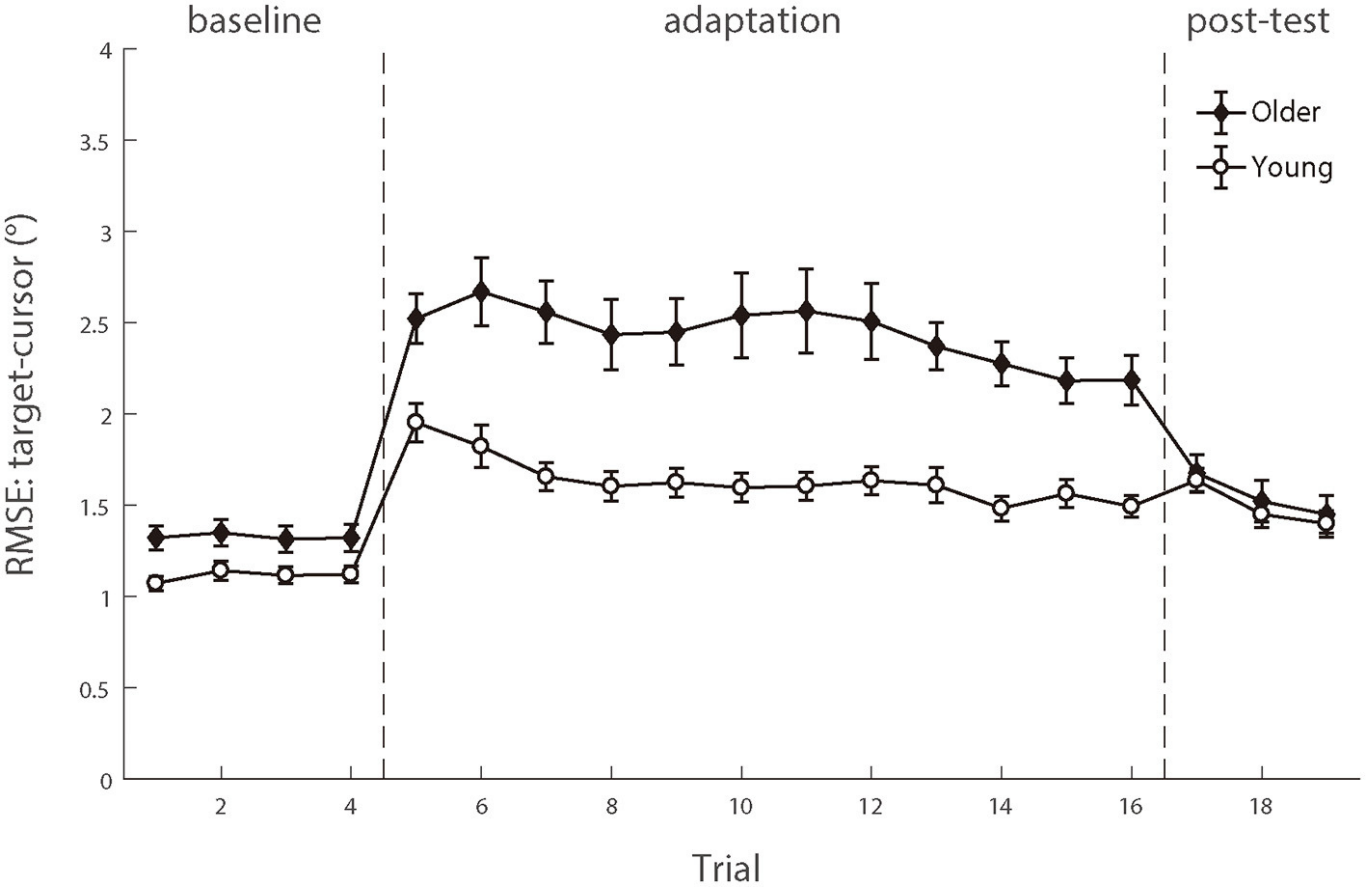
The means of RMSE between target and cursor after controlling for the lag effect as functions of trial order of young adults (white circle) and older people (black diamond). Positive lags represent that the cursor lags behind the target. Error bars depict ±1 standard errors of means.

To compare the random noise of visuomotor control between the two groups, we also analyzed the mean RMSE between the target and the cursor after controlling for the *target-cursor lag* effect. As shown in Figure 8, there were still significant main effects of age group condition [*F* (1.50) = 14.158, *p* < 0.001, *η_p_^2^* = 0.221] and test phase [*F* (4.200) =55.098, *p* < 0.001, *η_p_^2^* = 0.524], as well as a significant interaction between age group and post-test phase [*F* (4.200) = 12.672, *p* < 0.001, *η_p_^2^* = 0.202] (see the Supplementary Table A3). Then, we compared the mean differences between older adults and young adults during those three phases. There were significant age differences in the baseline phase [0.21 ± 0.09°, *p* = 0.017] and adaptation phase [0.80 ± 0.19°, *p* < 0.001], while no age group difference in the post-test phase [0.05 ± 0.13°, *p* = 0.682]. The interaction between the baseline and adaptation phase [*F* (1.50) = 14.415, *p* < 0.001, *η_p_^2^* = 0.224] was less than the interaction between the adaptation and post-test phase [*F* (1.50) = 26.163, *p* < 0.001, *η_p_^2^* = 0.344]. After controlling for the group differences in the baseline phase, a repeated measure ANOVA revealed a significant group effect over the adaptation phase [*F* (1.50) = 14.415, *p* < 0.001, *η_p_^2^* = 0.224], indicating that visual feedback perturbation resulted in not only longer *target-cursor lag* but also more increase of *random errors* in visuomotor control for the older adults (1.11 ± 0.13°) than for the young adults (0.52 ± 0.05°).

We also performed a linear mixed model to examine whether *random error* could explain the increased *target-cursor lag* with aging in manual tracking. Four alternative models were performed (see Table 2), which all included a control variable, *trial order*, which represented the order of a certain trial and was related to the general learning effect during the adaptation. We then tested two variables, *age* and *random error*, which represented the ages of individual participants and measured RMSE between target and cursor after controlling the *target-cursor lag* effect in specific trials, respectively. The model comparison (Table 2) showed that Model 4 which included *random error* had significantly better predictions than Model 1, χ^2^ = 291.58, *df* = 1, *p* < 0.001, and the fitting indices also showed a preference for Model 4 over Model 1. These results indicated that *random error* was also an effective predictor of *target-cursor lag*, even after the factor of individual ages had been controlled. On the other hand, there was no significant prediction difference between Model 4 and Model 5, in which the variable *age* was removed, χ^2^ = 0.083, *df* = 1, *p* = 0.773. Moreover, the fitting indices both showed a preference for Model 4 over Model 5. In the end, Model 6 which included both *random error* and *gaze-target lag* as predictors had significantly better predictions than Model 5 and Model 3, χ^2^ = 207.82, *df* = 4, *p* < 0.001, χ^2^ = 177.94, *df* = 4, *p* < 0.001, respectively. Considering these findings, we concluded that a combination of *gaze-target lag* and *random error* had the best prediction on degraded adaptation to delayed visual feedback in manual tracking, indicating that the age-related decreased function of visuomotor adaptation was a consequence of the combined effect from the declined capability of motor anticipation and the deterioration of motor execution.

**Table 2:** Linear mixed model comparison.

**Model**	**df**	**Log Likelihood**	**Deviance**	**AIC**	**BIC**
Model 1: TC_lag ~ age + TO	7	−1982.4	3964.9	3978.9	4005.8
Model 4: TC_lag ~ age + TO + RE	8	−1836.6	3673.3	3689.3	3720.1
Model 5: TC_lag ~ TO + RE	7	−1836.7	3673.4	3687.4	3714.3
Model 3: TC_lag ~ TO + GT_lag	7	−1821.7	3643.5	3657.5	3684.5
Model 6: TC_lag ~ TO + RE + GT_lag	11	−1732.8	3465.5	3487.5	3529.9

AIC, Akaike information criterion; BIC, Bayesian information criterion. TC_lag, target-cursor lag; TO, trial order; RE, random error; GT_lag, gaze-target lag.

**Figure 9:**
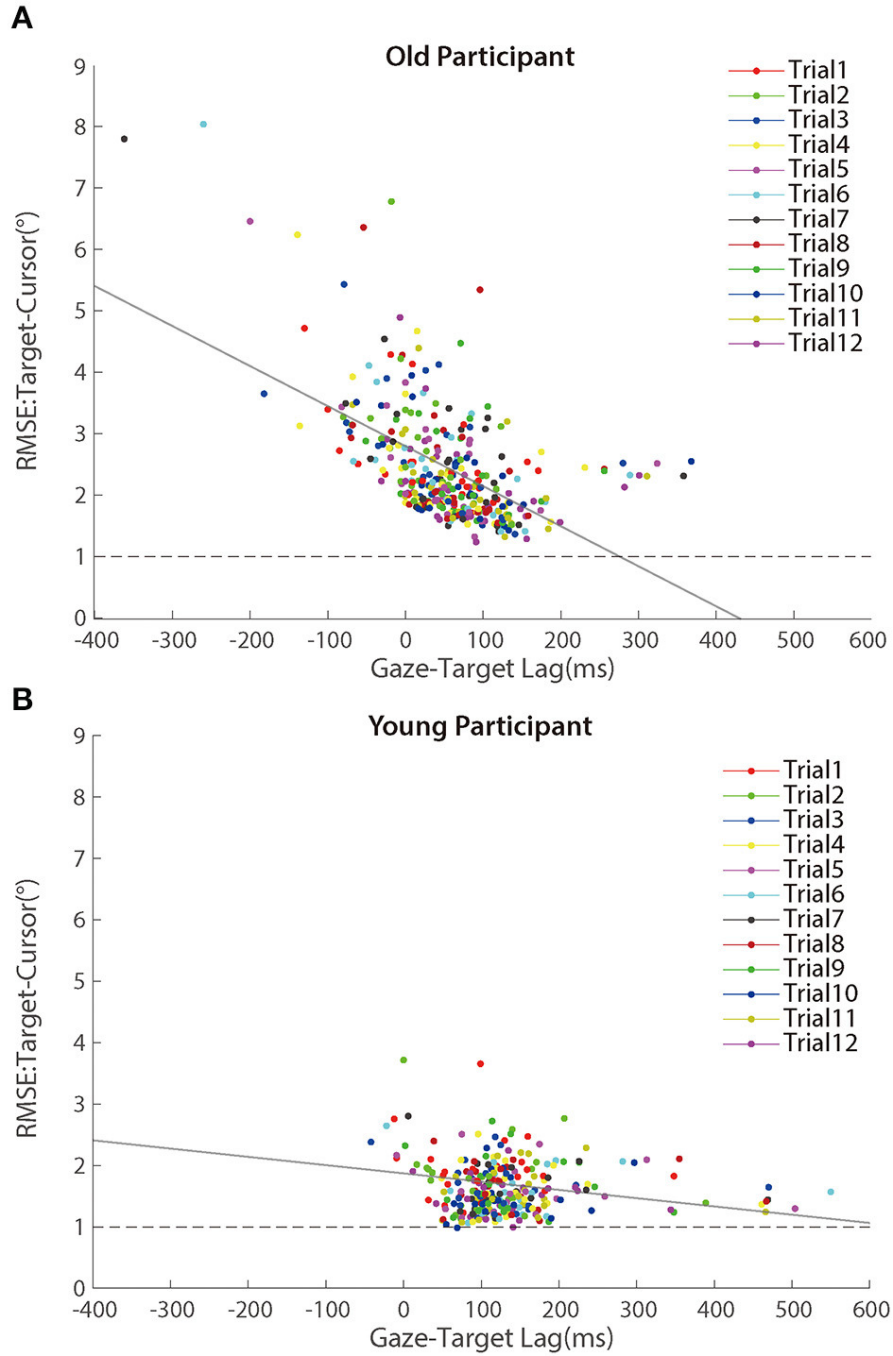
Scatter plots of RMSE between target and cursor after controlling for the lag effect in the adaptation phase for **(A)** older participants and **(B)** young participants. Trials in the same order are marked using a unique color. The solid lines represent simple linear regression model fits.

Further analyses revealed that the correlation between *random error* and *gaze-target lag* was a significant negative during the adaptation phase (Figure 9). We found significant correlations between age and *random error* in older adults [*r* (346) = 0.163, *p* = 0.002] and young adults [*r* (274) = 0.119, *p* = 0.049], respectively. Then, we tested the relationship between the *gaze-target lag* and the *random error* and found a mild negative correlation in older adults [*r* (346) = −0.553, *p* < 0.001], as well as a small correlation in young adults [*r* (274) = −0.120, *p* = 0.047].

To test whether the within-participant variations contributed to the negative correlation between *random error* and *gaze-target lag*, we re-calculated the correlations with centralized *random error* and *gaze-target lag* as before. The correlations did not change much for either group [older people: *r* (346) = −0.457, *p* < 0.001; young adults: *r* (274) = −0.164, *p* = 0.006], confirming the correlation between within-participant variations of *random error* and *gaze-target lag*.

## Discussion

As a summary of the results from the current study, the old participants' worse performance in visuomotor adaptation was related to both declined capability of motor anticipation and deterioration of motor execution. Motor anticipation measured by *gaze-target lag* showed a significant decrease for the older participants, and the differences in *gaze-target lag* across individual participants considerably predicted their performances in visuomotor adaptation, especially for the older participants. On the other side, we also found a significant deterioration of motor execution, measured by *random error* after controlling for the lag between target and cursor, for the older participants. Such deterioration was also significantly correlated with the declined performances in visuomotor adaptation, suggesting its influences on the age-related decline of visuomotor adaptation. These findings demonstrate that the age-related decline of visuomotor adaptation is a result of both declined capability of motor anticipation and deterioration of motor execution of older people.

### Visuomotor adaptation and aging

Throughout life, the human brain continually predicts and calibrates visuomotor errors. Our results showed that older people were able to adapt to a constant delayed visual perturbation in a predictable way as young people (Rohde et al., 2014). However, the results also indicated that this function declined with aging, demonstrated by insufficient adaptation to and more noisy responses triggered by delayed visual feedback of the older participants, compared to the younger ones. These findings were consistent with the previous studies which measured visuomotor adaptation using other tasks, including prism adaptation task (Fernández-Ruiz et al., 2000), mirror-tracing/spatial reversal adaptation tasks (Bock and Schneider, 2002; Rodrigue et al., 2005), and center-out rotation adaptation tasks (Buch et al., 2003; Bock, 2005; Bock and Girgenrath, 2006; Seidler, 2006; Heuer and Hegele, 2008; Vandevoorde and de Xivry, 2019; Wolpe et al., 2020; Li et al., 2021). For instance, in the prism adaptation task, where participants were asked to point at the targets with prism goggles that displaced the visual field laterally, the aged group showed a slower visuomotor adaptation than the young group (Fernández-Ruiz et al., 2000). Similarly, researchers found from mirror-tracing tasks that older participants had slower adaptation (Rodrigue et al., 2005) and larger RMSE between target and cursor (Bock and Schneider, 2002) than young adults. Such effects were also widely observed from rotation adaptation tasks, the most commonly used paradigm for studying visuomotor adaptation (Buch et al., 2003; Bock, 2005; Bock and Girgenrath, 2006; Seidler, 2006; Heuer and Hegele, 2008; Vandevoorde and de Xivry, 2019; Wolpe et al., 2020; Li et al., 2021). Our new observations are generally consistent with these findings but have also extended the literature by showing that the declined capability of visuomotor adaptation could also occur in a real-time online control task. From this perspective, this age-related decline is not specific to tasks but is due to a degraded function of visuomotor control with advancing age.

### The mechanisms of the age-related deficits in visuomotor adaptation

A critical purpose of the current study was to investigate what mechanisms underlie age-related decline in visuomotor adaptation. As mentioned in the Introduction section, there are two mechanisms, the deteriorations of motor anticipation and of motor execution, which possibly underlie the decline in visuomotor adaptation. Motor anticipation, also known as “prediction” (Bubic et al., 2010) or a part of “motor planning” (Svoboda and Li, 2018), refers to one's capability of predicting or planning future actions and this capability can help to adapt changes of the mapping between input from outside and internal representation. This capability is also closely related to general cognitive functions (Chen et al., 2022), which decline gradually with aging (Salthouse, 1996; Raz, 2000; Park et al., 2003) and is found to be a mediator between aging and decline of visuomotor adaptation (Vandevoorde and de Xivry, 2019, 2020; Wolpe et al., 2020; Li et al., 2021). The other possible mechanism, the deterioration of motor execution, refers to the deterioration of real-time online control of actions. Previous studies have shown that motor execution of older people is either delayed (Bock and Schneider, 2002; Teulings et al., 2002) or becomes less accurate (Buch et al., 2003; Bock, 2005; Bock and Girgenrath, 2006; Seidler, 2006; Heuer and Hegele, 2008; Vandevoorde and de Xivry, 2019; Wolpe et al., 2020; Li et al., 2021), indicating the relationship between its deterioration and aging.

To distinguish the contributions of two mechanisms to the age-related decline of visuomotor adaptation, we chose a continuous tracking task and recorded the participants' eye movement during their tracking. The continuous tracking task required the participants to keep making short-term predictions of object motions and planning hand movements (i.e., motor anticipation) all the time during the task, and the recorded eye movements provided a window to investigate how motor anticipation was exactly made and modulated with visuomotor adaptation (Ariff et al., 2002; Barnes, 2008; Kettner et al., 1997; Rand and Rentsch, 2016; Brouwer et al., 2018; Kowler et al., 2019). In the current experiment, we found that the recorded gazes always preceded the target and such predictive pursuit eye movements reflected individuals' predictive processes about future motions of the target during tracking, as found in previous studies (Kettner et al., 1997; Barnes, 2008; Kowler et al., 2019). More interestingly, we found that the older participants showed a significantly shortened *gaze-target lag* (see Figure 6B), indicating the decline of the predictive processes with aging. This result was consistent with Maruta et al. (2017) study in which a sample of 143 participants (age range: 7–82 years) was included and they found that the positional precision and smooth pursuit velocity gain of visual tracking declined over age when people were over the age of 50 years. In addition, our result was also consistent with the previous studies using manual tracking tasks (Welford, 1958; Braune and Wickens, 1985; Jagacinski et al., 1993), in which older people were found to perform worse when they needed to overcome sensory-motor delays and had longer lags behind the target. This decline in *gaze-target lag* was also strongly correlated with the increase in *target-cursor lag*, especially in the older participants (*r* = −0.74). Regarding the tight relationship between motor anticipation and predictive pursuit eye movements (Ariff et al., 2002; Barnes, 2008; Kettner et al., 1997; Rand and Rentsch, 2016; Brouwer et al., 2018; Kowler et al., 2019), this observed strong correlation between predictive pursuit eye movements and visuomotor adaptation further indicated that the declined capability of motor anticipation was possibly a critical factor in the declined performance of a visuomotor adaptation of the older participants. In other words, older people's worse performances in visuomotor adaptation were likely to be due to their declined capacity of motor anticipation.

The effect of declined motor anticipation on visuomotor adaptation is also consistent with the previous findings from studies involving ballistic reaching tasks. Although there are mixed results from empirical studies on how aging affects visuomotor adaptation, a consistent and important finding across the existing studies is that aging mainly affects explicit components of visuomotor adaptation in ballistic reaching tasks (Heuer and Hegele, 2008; Hegele and Heuer, 2010a, 2013; Vachon et al., 2020). In addition, such age-related decline of visuomotor adaptation is tightly related to the deterioration of explicit memory, as shown in studies by Li et al. (2021) and Wolpe et al. (2020). Considering that motor anticipation is generally associated with explicit processing (Varghese et al., 2016; Svoboda and Li, 2018; Chen et al., 2022), our results further confirm that aging mainly results in the decline of explicit components of visuomotor control. Common mechanisms might be shared by the age-related effects on the adaptation of ballistic reaching tasks and online manual tracking tasks.

In addition to testing how declined motor anticipation affected visuomotor adaptation, we examined whether the deterioration of motor execution also contributed to worse performance in the visuomotor adaptation of older people. To distinguish the contributions of motor execution and motor anticipation, we tested *random error* of hand movements after controlling for systematic variation by *target-cursor lag*. The remaining *random error* was used as a measure of motor execution. In the current study, the increase of *random error* caused by feedback was significantly higher for the older participants (see Figure 8) and, as shown by the linear mix model analysis, this increased *random error* was related to the increase of *target-cursor lag*. These results both indicated that the deterioration of motor execution might be another important factor in the declined performance of a visuomotor adaptation of the older participants. More interestingly, this increase in *random error* was moderately correlated with the decrease in *gaze-target lag*, especially in the older participants (*r* = 0.55). This correlation suggests that both mechanisms underlying visuomotor adaptation may share some common neural substrates, which are further discussed in the following subsection.

However, one may note that errors in motor execution can be caused by different mechanisms, including a worse online correction to visual feedback, worse movement selection, and worse simple movement execution. In the current study, the participants needed to continuously respond to dynamic changes of the target during the manual tracking task, and the random errors in their performances were apparently from the collective influences of these potential mechanisms, which might limit us to distinguish these mechanisms and their independent contributions. For example, it was difficult to quantify the online correction to a certain visual perturbation with the current task as in the previous studies (e.g., Körding and Wolpert, 2004; Saunders and Knill, 2004; Greenwald et al., 2005) because visual perturbations were continuously presented during the whole tracking procedure in the current study. Such an online perturbation method is possibly used to compare the capability of online correction to visual updates between older and young people in future research. On the other side, we think that the declined stability of simple movement execution might have a significant but small contribution to the aging effect on visuomotor adaptation because of a finding from our previous study (Li et al., 2021). In that study, we conducted a ballistic reaching task and observed a small age-related effect on random error (older people: 5.41° ± 0.15° *s.e., N* = 100; young adults: 4.99° ± 0.27° *s.e., N* = 20) in the conditions without perturbation. Note that Li et al. (2021) only reported the significance of this effect but not the mean values. Similar effects have been found in other previous studies (Hegele and Heuer, 2010a,b) as well. In addition, the smaller age-related difference of lag-corrected random error in the baseline trials, compared to the larger effect in the adaptive trials, of the present study also indicates that the decline of simple movement execution by aging was limited, though significant. In short, we speculate that the observed age-related difference in lag-corrected random error in this study was possibly a combined effect of declined capability of online correction, and declined stability of simple movement execution, between which declined capability of online correction might have a major contribution.

In addition to analyzing eye–hand coordination, one can also investigate individuals' mental processing by analyzing other oculomotor or pupillary responses. For example, multiple studies have shown that the frequency of saccadic eye movements during pursuit is negatively correlated with an individual's cognitive functions (Galna et al., 2012; Stuart et al., 2018). In the current study, we compared the numbers of saccades in each trial between two groups and found a significant increase for the older group. Although the current experiment conducted a manual tracking task rather than a pursuit task, previous studies have indicated that the two tasks have shared mechanisms (Engel et al., 2000; Niehorster et al., 2015), and we considered that the increased saccadic eye movement during manual tracking of the older groups reflected their decreased cognitive functions, which were consistent with the previous findings (Ariff et al., 2002; Rand and Rentsch, 2016; Brouwer et al., 2018) and the observed decline of motor anticipation from this experiment. We also analyzed individuals' pupil size variations to investigate whether their arousal levels changed during the experiment (Stanners et al., 1979; Murphy et al., 2011; Jerčić et al., 2020). The results nevertheless showed that neither group showed significant pupil size change during the experiment, indicating that there was no marked variation in arousal level with the progress of the experiment for either group. An interesting finding regarding pupil size was that the older group had a significantly smaller mean pupil size than the young group. This finding was consistent with previous literature (Birren et al., 1950; Telek et al., 2018). However, we did not record actual pupil sizes but only ones measured by pixels on the tracker camera. To make between-participant comparisons in actual pupil size, one would need to further measure the size of an artificial pupil as a reference, which was not measured in the current experiment. So, we chose to not conclude this finding.

### Shared neural substrates of aging effects on motor anticipation and execution?

As mentioned in the Introduction section, the corresponding neural substrates of motor anticipation and motor execution were usually considered to be differentiated. For instance, Glover et al. (2012) separated the visual cortical networks into a planning network, which includes the premotor cortex, basal ganglia, anterior cingulate, posterior medial parietal area, superior parietal occipital cortex, and middle intraparietal sulcus, and an online control network which includes the sensorimotor cortex, cerebellum, supramarginal gyrus, and superior parietal lobule. The former network, especially the premotor, frontoparietal, and occipitotemporal cortices, are typically involved in the anticipatory processing of motor control (Bubic et al., 2010; Diersch et al., 2013). Our findings suggest that motor anticipation and motor execution both decline with aging and contribute to age-related declined performances of visuomotor adaptation. These are consistent with previous works showing that all those related brain regions had age-related atrophy, including frontal lobes (Resnick et al., 2003; Raz, 2005), parietal lobes (Resnick et al., 2003; Raz, 2005), basal ganglia (Hubble, 1998), cerebellum (Raz et al., 2001, 2005; Raz, 2005; Filip and Bare, 2021), and so forth.

More interestingly, we found that aging effects related to motor anticipation and motor execution had a moderate correlation with each other. This finding suggests that these two mechanisms may share some same neural substrates, which is consistent with the previous findings that some neural substrates are involved in motor anticipation and motor execution. For example, the cerebellum, which was once considered to be exclusively linked to motor execution, is involved in predicting events about motor timing perceptual (O'Reilly et al., 2008; see review in Flesischer, 2007) and coding of future movement in the frontal cortex (Gao et al., 2018). Another case is the basal ganglia which are considered to play a critical role in making adaptive anticipation of and correcting ongoing movements (Tunik et al., 2009; see review in Flesischer, 2007), while the cortico-basal ganglia circuitry shows aberrant during motor task execution (Taniwaki et al., 2007; Marchand et al., 2011).

Our study extends its findings from a behavioral perspective by providing new evidence that age-related declines of motor anticipation and motor execution both feature in the cases of visuomotor adaptation. The similarity in neural substrates among the two motor-related processes and the aging effects on both of them suggests that the declined performances of visuomotor adaptation with aging reflect a general deterioration of the motor-related system in older people and support the previous suggestions in studies (Milton et al., 1989; Beuter et al., 1990) that manual tracking with delayed visual feedback could potentially be used as a clinical method for screening motor-related disorders. Nevertheless, the current study did not measure any neural signals directly, limiting us to make any conclusion about the relationship between different types of motor control and their underlying neural mechanisms. In future research, we plan to introduce neural approaches (e.g., recording neural oscillations) to explore underlying neural mechanisms of the aging effect on visuomotor adaptation.

## Conclusion

In summary, our study provides evidence that both declined capability of motor anticipation and deterioration of motor execution contributed to the age-related decline of visuomotor adaptation. Motor anticipation, measured by gaze-target lag, showed a significant decrease in older participants which then predicted the decline of older individuals' performances in visuomotor adaptation. Our data also reveal a significant deterioration of motor execution for older participants, measured by random error after controlling for the lag between the target and the cursor. The mixed linear model analysis also showed that a combination of gaze-target lag and random error had the best prediction on degraded adaptation to delayed visual feedback in manual tracking, indicating the significant roles of both the declined capability of motor anticipation and the deterioration of motor execution in the age-related decline of visuomotor adaptation. These findings are broadly consistent and have extended the existing literature on the mechanisms underlying age-related declines in visuomotor adaptation, helping to understand the emergence of motor-related dysfunctions with aging. This study provides a basis for future research on the interventions to address the degraded motor control of older people.

## Data availability statement

The datasets presented in this study can be found in online repositories. The names of the repository/repositories and accession number (s) can be found below: https://osf.io/tq8ye.

## Ethics statement

The studies involving human participants were reviewed and approved by Committee on Human Research Protection, East China Normal University. The patients/participants provided their written informed consent to participate in this study.

## Author contributions

NL and ZC developed the conceptual framework and conceived and designed the experiments. NL and YX programmed and performed the experiments. NL, YX, and ZC analyzed the data. NL, JL, WJ, and ZC wrote the manuscript. All authors contributed to the article and approved the submitted version.
