# Supplementary material for: Age-related decline of online visuomotor adaptation: a combined effect of deteriorations of motor anticipation and execution
Source: Front Aging Neurosci. 2023 Jun 20;15:1147079. doi: 10.3389/fnagi.2023.1147079 (PMC10318141; doi:10.3389/fnagi.2023.1147079)
Supplement: Supplementary file 1 [file Data_Sheet_1.pdf]

Appendix

Table A1. ANOVA results: RMSE between target and cursor for the older and young participants during 5 test phase conditions. Test phase conditions included baseline (Base), adaptation phase1 (Adapt1), adaptation phase2 (Adapt2), adaptation phase3 (Adapt3) and post-test (Post-test).

Two-way repeated-measures ANOVA with factors (RMSE)			
Test phase	5 levels:		
	Base, Adapt1, Adapt2, Adapt3, Post-test		
Age group	2 levels: young, older		
Test phase	F (4,200) = 103.604	$p < 0.001$	$Eta = 0.674$
Age group	F (1,50) = 22.747	$p < 0.001$	$Eta = 0.313$
Test phase × Age group	F (4,200) = 12.338	$p < 0.001$	$Eta = 0.198$
Base-Adapt1	−1.123±0.073	$p < 0.001$	
Adapt1-Adapt2	0.165±0.051	$p = 0.021$	
Adapt2-Adapt3	0.195±0.073	$p = 0.100$	
Adapt3-Post-test	0.636±0.055	$p < 0.001$	
Base-Post-test	−0.127±0.04	$p = 0.026$	
Adapt1-Adapt3	0.359±0.058	$p < 0.001$	

Table A2. ANOVA results: the latency between target and cursor for the older and young participants during 5 test phase conditions.

Two-way repeated-measures ANOVA with factors (target-cursor lag)			
Test phase	5 levels:		
	Base, Adapt1, Adapt2, Adapt3, Post-test		
Age group	2 levels: young, older		
Test phase	F (4,200) = 103.816	$p < 0.001$	$Eta = 0.675$
Age group	F (1,50) = 27.08	$p < 0.001$	$Eta = 0.351$
Test phase × Age group	F (4,200) = 8.687	$p < 0.001$	$Eta = 0.148$
Base-Adapt1	−107.592±7.909	$p < 0.001$	
Adapt1-Adapt2	28.533±7.295	$p = 0.003$	
Adapt2-Adapt3	26.155±10.7	$p = 0.181$	

Adapt3-Post-test	94.582±5.935	$p < 0.001$
Base-Post-test	41.677±5.22	$p < 0.001$
Adapt1-Adapt3	54.687±7.756	$p < 0.001$

Table A3. ANOVA results: random error for the older and young participants during 5 test phase conditions.

Two-way repeated-measures ANOVA with factors (random error)			
Test phase	5 levels:		
	Base, Adapt1, Adapt2, Adapt3, Post-test		
Age group	2 levels: young, older		
Test phase	F (4,200) = 55.098	$p < 0.001$	*Eta* = 0.524
Age group	F (1,50) = 14.158	$p < 0.001$	*Eta* = 0.221
Test phase×Age group	F (4,200) = 12.672	$p < 0.001$	*Eta* = 0.202
Base-Adapt1	−0.933±0.08	$p < 0.001$	
Adapt1-Adapt2	0.087±0.053	$p = 1$	
Adapt2-Adapt3	0.170±0.077	$p = 0.318$	
Adapt3-Post-test	0.373±0.057	$p < 0.001$	
Base-Post-test	−0.302±0.041	$p < 0.001$	
Adapt1-Adapt3	0.257±0.058	$p = 0.001$	
